# Understanding, diagnosing, and treating pancreatic cancer from the perspective of telomeres and telomerase

**DOI:** 10.1038/s41417-024-00768-6

**Published:** 2024-04-09

**Authors:** Songting Shou, Yuanliang Li, Jiaqin Chen, Xing Zhang, Chuanlong Zhang, Xiaochen Jiang, Fudong Liu, Li Yi, Xiyuan Zhang, En Geer, Zhenqing Pu, Bo Pang

**Affiliations:** 1 Guang’anmen Hospital, China Academy of Chinese Medical Sciences, Beijing, China; 2 Department of Oncology, The First Affiliated Hospital of Guangzhou University of Chinese Medicine, Guangzhou, China; 3 Department of Gastroenterology, Dongzhimen Hospital, Beijing, China

**Keywords:** Biomarkers, Cancer

## Abstract

Telomerase is associated with cellular aging, and its presence limits cellular lifespan. Telomerase by preventing telomere shortening can extend the number of cell divisions for cancer cells. In adult pancreatic cells, telomeres gradually shorten, while in precancerous lesions of cancer, telomeres in cells are usually significantly shortened. At this time, telomerase is still in an inactive state, and it is not until before and after the onset of cancer that telomerase is reactivated, causing cancer cells to proliferate. Methylation of the telomerase reverse transcriptase (TERT) promoter and regulation of telomerase by lactate dehydrogenase B (LDHB) is the mechanism of telomerase reactivation in pancreatic cancer. Understanding the role of telomeres and telomerase in pancreatic cancer will help to diagnose and initiate targeted therapy as early as possible. This article reviews the role of telomeres and telomerase as biomarkers in the development of pancreatic cancer and the progress of research on telomeres and telomerase as targets for therapeutic intervention.

## Introduction

Pancreatic cancer is currently the twelfth most common cancer and the seventh most common cause of cancer-related death worldwide [1]. According to statistics in recent years, the number of deaths and the incidence of pancreatic cancer in the world are very close, indicating that the prognosis of the disease is very poor [1]. Pancreatic cancer is usually considered resectable if there is minimal contact with major vessels [2]. If a patient has advanced pancreatic cancer, gemcitabine and albumin combined with paclitaxel or modified FOLFIRINOX are often used as first-line treatment [3, 4]. However, by the time the disease is diagnosed, patients are often in a late stage disease and treatment response is less than ideal [2]. The five-year survival rate is less than 10% [5]. Therefore, researchers have initiated multidisciplinary and interdisciplinary research on the treatment and disease management of pancreatic cancer to help patients improve their quality of life [6]. The pathogenesis of pancreatic cancer still needs further exploration, especially in the fields related to other pancreatic diseases. Many pancreas-related diseases increase the risk of pancreatic cancer, such as hereditary pancreatitis, chronic pancreatitis, diabetes, etc [7–9]. These chronic diseases will lead to cell aging and promote pancreatic cancer through some mechanisms that are not yet fully understood.

**Fig. 1:**
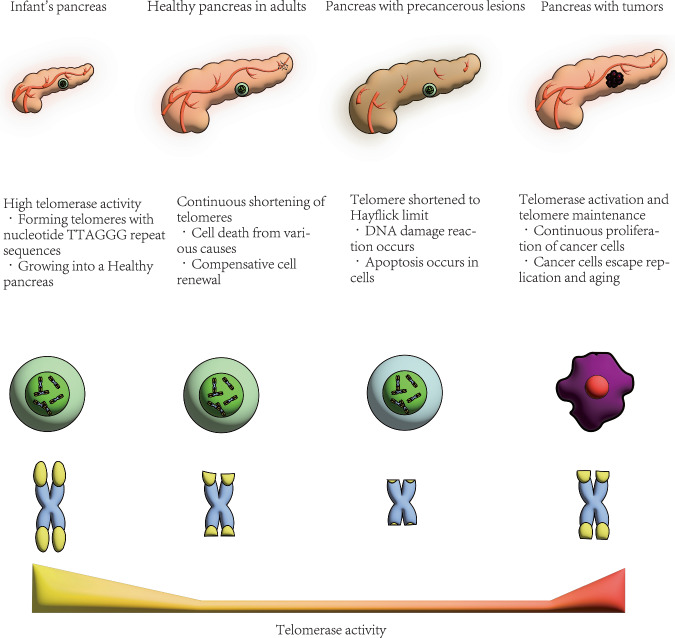
Telomerase presence and telomere maintenance in the pancreas. The development of telomerase expression and telomere length are displayed in the context of health and disease. Telomeres are the DNA Protein structure at the end of chromosomes. Telomeres are synthesized by telomerase, and the activity of telomerase in the infant’s pancreas is strong, which promotes the proliferation of healthy pancreatic cells. In adulthood or after illness, exposure to adverse sleep habits, diseases, and other risk factors can induce inflammation, oxidative stress, and cell death [155]. Subsequently, the pancreas maintains its function through cell renewal, but this process can lead to telomere shortening. The shortening of telomeres to a certain length may be the fundamental reason for cell division reaching the Hayflick limit, during which cells undergo replication aging, leading to DNA damage response (DDR) and cell senescence. Before and after pancreatic cancer, telomerase is reactivated, and telomeres are maintained at a certain length, which allows pancreatic cancer cells to escape from replicative senescence and make pancreatic cancer continue to develop.

It has been known for decades that cancer is usually the result of changes in somatic chromosomes [10–13]. Using next-generation RNA sequencing, researchers found that on average there are 63 mutated genes per tumour. Most of these mutations are point mutations and affect the core set of 12 cell signalling pathways in most tumours, such as apoptosis, DNA damage control, regulation of G1/S phase transition, hedgehog signaling, or other pathways that produce effects such as homophilic cell ashesion, etc [14]. The study showed that the role of the mutated genes varies greatly in different tumours, making targeted personalised therapy essential. A review of 39 studies found that telomerase activity can help doctors differentiate between pancreatitis and pancreatic cancer, and that telomerase plays a key role in malignant cell immortality [15]. Telomeres and telomerase can maintain cell and human vitality, but in cancer cells, they can be detrimental to human health. This means that it can assist tumour cells to proliferate and, because of the nature of tumour cells, their ability to proliferate exceeds that of normal cells, leading to cancer and even death (Fig. 1). In this review, we describe the changes in telomeres and telomerase in different stages of pancreatic cancer. We also introduce the biomarkers associated with telomeres, telomerase and pancreatic cancer, and how telomerase may be targeted for the treatment of pancreatic cancer in the future.

## Function and structure of telomere and telomerase

**Fig. 2:**
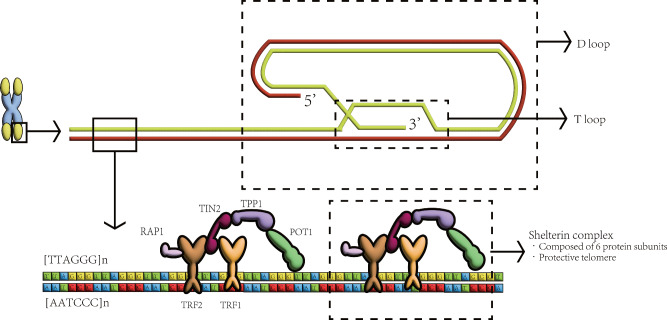
The vertebrate telomere complex. The telomere has a T-loop and D-loop structure, which is divided into 3 ‘and 5’ ends at the end. There is a Shelterin complex on the telomeres, which helps maintain stability. POT1 protection of telomeres 1. RAP1 repressor/activator protein 1. TIN2 TERF1-interacting nuclear factor 2. TPP1 telomere protection protein 1. TRF1 telomeric repeat binding factor 1. TRF2 telomeric repeat binding factor 2.

In mammalian cells, including humans, telomere DNA is composed of double-stranded tandem repeats containing TTAGGG, comprising thousands to tens of thousands of bases [16]. These double strands form a loop structure, called a T loop, with a 3’G-rich single-stranded overhang at the end that inserts into the double-stranded DNA and returns to the telomere to form the so-called D loop [17] (Fig. 2). Telomeres are associated with 6 protein subunits, TRF1, TRF2, TPP1, POT1, TIN2 and RAP1, which form a complex called the shelterin complex [18] (Fig. 2). This structure protects telomeres and inhibits telomere damage, while telomeres can protect DNA and prevent DNA from being damaged [18]. In addition to protecting DNA, telomeres can also regulate the pluripotency of stem cells and maintain their homeostasis [19]. All replicating or dividing human somatic cells shorten their telomeres due to the end replication problem (ERC) due to the semiconservative mechanism of DNA replication [20]. Once the telomeres in a cell have shortened to a certain length, the cell begins to undergo replicative senescence or programmed death [21]. All this suggests that the structure and function of telomeres are closely linked to disease, aging and other life processes. By 1999, researchers had established a link between cancer and telomeres [22].

**Fig. 3:**
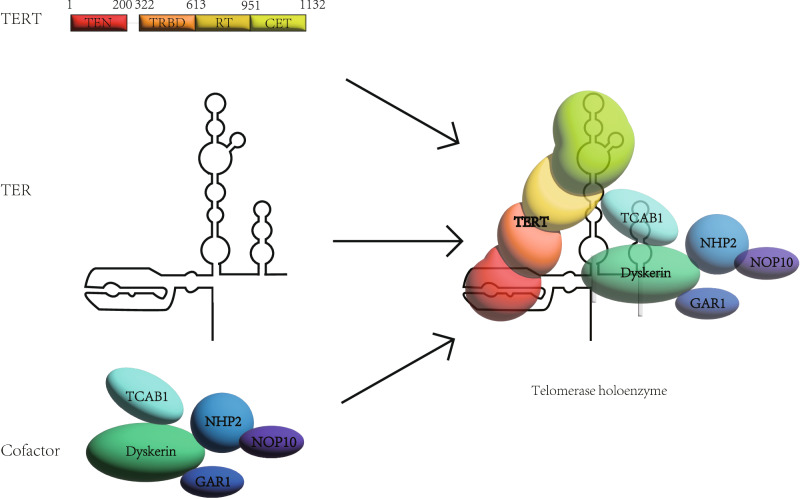
The vertebrate telomerase complex. The composition of human telomerase is divided into three parts: TERT, TEN, and Cofactor. They combine together to form telomerase, maintaining the length of telomeres. GAR1 nucleolar protein family A. member 1. NHP2 nucleolar protein family A, member 2. NOP10 nucleolar protein 10. TCAB1 telomerase Cajal body protein 1.

Human telomerase is mainly composed of two parts, one is called telomerase RNA (TER), and the other is called TERT [23] (Fig. 3). The activity of telomerase mainly depends on the expression of TERT [24]. The human TERT is called hTERT. The conservative domain of TERT has four parts, which are arranged in a linear manner and maintain its biological function. One end is the telomerase essential N-terminal (TEN) domain, the other end is the C-terminal extension (CTE) domain, and the TER binding domain (TRBD), the reverse transcriptase (RT) domain between them [25]. In addition to TERT and TER, the composition of cell telomerase holoenzyme is more complex. The telomerase holoenzyme also includes some cofactors, which have an important impact on the function of telomerase [26–28]. The main function of telomerase is to synthesize telomeres, which can maintain a stable length due to the existence of telomerase [29, 30].

## Telomeres and precancerous lesions of pancreatic cancer

Pancreatic intraepithelial neoplasia (PanIN) is the most common precursor lesion of pancreatic cancer [31]. In addition, there are two common precursor lesions, namely mucinous cystic neoplasm (MCN) and intraluminal papillary mucinous neoplasm (IPMN) [32]. In PanIN and IPMN, there are many changes in the epithelial cells affected by the disease, one of which is the presence of telomere shortening [33, 34]. Other studies have shown that the telomere-centromere ratio (TRC) decreases as the pathological grade of IPMN increases [35]. This suggests that telomeres become progressively shorter as the disease progresses. Telomere shortening is the most common early genetic abnormality in current pancreatic cancer models, but this telomere shortening is not the direct cause of cancer. The direct cause is likely to be that telomere shortening leads to chromosomal abnormalities, such as fusion of chromosomes, or deletion and insertion of a particular part of the chromosome [36]. The presence of unbalanced chromosomal rearrangements is a necessary condition for most human epithelial cancers [36]. Some studies have shown that many chromosomes in pancreatic cancer often have various abnormalities [37, 38]. Many other studies have linked telomere dynamics to aging, demonstrating that inflammation, epigenetic dysregulation, loss of proteostasis, cellular senescence, stem cell exhaustion, mitochondrial dysfunction caused by telomere dysfunction are almost all signs of aging that will lead to the onset or acceleration of aging [39]. Age-related morphological changes of the pancreas are closely related to the occurrence of pancreatic cancer [40]. Therefore, the development of pancreatic cancer is likely to be caused by age-related telomere shortening and subsequent telomere dysfunction, leading to chromosomal instability (CIN), chromosomal abnormalities and consequently the development of pancreatic cancer [41–43]. Some studies suggest that cancer stem cells originate from differentiated tumor cells [44–46]. Some researchers have proposed other pathogenic models, suggesting that the onset of cancer occurs due to abnormalities in the self-renewal of normal stem cells, leading to the emergence of tumor stem cells that exhibit increased resistance to drug therapy and apoptosis [47]. This phenomenon partly explains the ineffectiveness of standard chemotherapy and radiotherapy in early-stage pancreatic cancer. It is speculated that this phenomenon may be associated with telomeres and telomerase in pancreatic cancer stem cells (PCSCs). A study reveals that pancreatic cancer stem cells exhibit an elevated telomerase activity in comparison to non-stem pancreatic tumor cells, concurrent with the elongated telomere lengths [48]. The initiation of telomerase function within PCSCs seems intrinsically connected to the aberrant expression of core pluripotency factors, such as SOX2, OCT3/4, KLF4, and NANOG, where the overexpression of any single constituent among these can instigate a collective upregulation of the remaining factors, thereby amplifying telomerase activity within these specific cell type [48]. Therefore, cancer stem cells may maintain their stemness through telomere and telomerase-related mechanisms, facilitating the progression from pancreatic cancer precursors to pancreatic cancer. This continuous proliferation may drive the transition of early-stage pancreatic cancer to advanced-stage pancreatic cancer.

## Special mechanism of telomerase reactivation in pancreatic cancer

### Chromosome changes

**Fig. 4:**
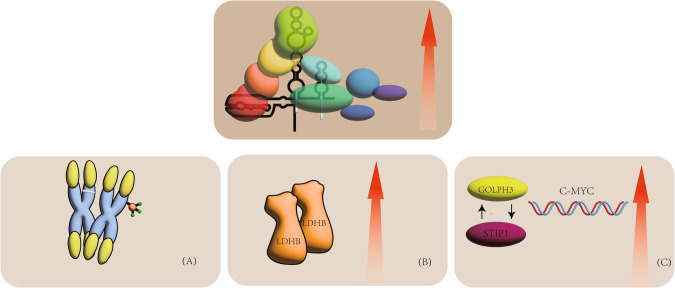
The activation mechanism of telomerase. **A** Loss or methylation of gene fragments on chromosomes. **B** Increase in LDHB protein. **C** The interaction between GOLPH3 and STIP1 increases the expression of c-MYC.

The TERT promoter is altered in many cancers, including melanoma, liposarcoma, hepatocellular carcinoma, hepatocellular carcinoma and urothelial carcinoma. However, some studies have found that mutations in the TERT promoter are rare in pancreatic cancer cases [49–51]. Different from other cancers, pancreatic cancer may originate from chromosome changes. In 2021, The research team of Yagyu et al. found that a gene in the 3p21.3 chromosome region inhibits TERT by inhibiting the activity of the TERT promoter, thereby inhibiting the invasion and proliferation of pancreatic cancer cells, thus achieving the effect of inhibiting cancer [24] (Fig. 4). The probability of detecting loss of heterozygosity in this region in other cancers has reached 75%, but it has not been established that this alteration is associated with the development of pancreatic cancer [24]. Therefore, at present, the development of pancreatic cancer may be due to a different mechanism, namely methylation of the TERT promoter. In 2009, an Indian study found that methylation of hTERT promoter and p16 promoter was positively correlated with hTERT expression and telomerase activity by comparing pancreatic cancer tissue with adjacent normal tissue [52]. The researchers in this study also found that the use of 5-azacytidine, a demethylating agent, in pancreatic tissue resulted in a decrease in hTERT gene expression, leading to a decrease in telomerase activity. Demethylation of 5-azacytidine leads to hTERT inhibition, which can reduce telomerase activity to 37–49% of the control group [53]. Therefore, it is likely that hTERT promoter methylation regulates the expression of hTERT and then regulates telomerase activity, thereby affecting the occurrence and development of pancreatic cancer.

Current research indicates that in a 433 bp genomic region known as the TERT hypermethylated tumor region (THOR), located at the 52 CpG sites upstream of the TERT core promoter. It has been observed that unmethylated THOR exerts an inhibitory effect on TERT promoter activity, whereas extensive methylation of THOR counteracts this inhibitory function [54]. Some researchers speculate that the THOR sequence may contain multiple binding sites for transcription inhibitors, such as WT1 and MZF2 binding sites [55]. In highly methylated THOR, these sites may be unable to inhibit factor binding, potentially leading to the abnormal upregulation of TERT [56]. Further research is needed to elucidate the precise mechanisms underlying this phenomenon.

### The presence of LDHB

The sustained proliferation of cancer cells is related to telomere length, which is usually associated with telomerase activity. The maintenance of telomerase activity requires certain conditions. In 2022, a cellular study conducted by Wang et al. showed that regardless of its metabolic pathway, LDHB is essential for maintaining telomere length and promoting telomerase activity [57] (Fig. 4). Knocking out LDHB can inhibit tumour cell proliferation, telomerase activity and reduce telomere length in both in vivo and in vitro experiments [57]. The team also found that LDHB was negatively correlated with the disease-free survival (DFS) of patients by detecting pancreatic cancer tissue and adjacent positive tissue [57]. This is somewhat different from the research of Cui et al. In 2015, Cui et al. showed that LDHB in pancreatic cancer tissues was higher than that in normal adjacent tissues, and inhibiting the expression of LDHB could promote the growth and development of pancreatic cancer [58]. This growth promotion may be caused by an increase in glycolysis, and LDHB is an important enzyme in this pathway. Previous studies have shown that LDHB is an important enzyme in the glycolytic pathway. LDHB can also activate tumor cell lysosomes and perform autophagy through lysosomes, thereby regulating metabolism when tumor cells are under nutrition [59]. However, Cui et al’s study found that the mechanism by which LDHB prolongs telomeres is mainly due to the direct interaction between LDHB and TERT to regulate telomerase, and no association has been found between LDHB and its role in glycolysis [57].

### The Interaction between GOLPH3 and STIP1

Golgi phosphoprotein 3 (GOLPH3) is a highly conserved cytosolic trans-Golgi associated protein [60]. The study by Sun et al. found that overexpression of GOLPH3 significantly increased the expression of β‐catenin, c‐Myc, and cyclin‐D1 [61]. However, this study was found in ovarian cancer cells, so it still needs to be further verified in pancreatic cancer cells. Stress-inducible protein-1 (STIP1) is a novel GOLPH3 binding partner [62]. STIP1 can promote the proliferation of pancreatic cancer cells and accelerate the cell cycle [62]. Telomerase activity is regulated by hTERT, which is activated by c-Myc through the E-box [63] (Fig. 4). Studies have shown that inhibition of hTERT can inhibit cyclin D1 signalling in cancer cells and also inhibit the cell cycle [64, 65]. When GOLPH3 and STIP1 are knocked down, they can not only inhibit the proliferation of pancreatic cancer cells, but also inhibit the expression of cyclin D1 in cancer cell [62]. This study also showed that the overexpression of GOLPH3 was positively correlated with the expression of STIP1 in pancreatic cancer tissue, but the knockout of GOLPH3 did not affect the level of STIP1 [62].

## Telomere and telomerase as biomarkers

Biological markers, also known as biomarkers, are objectively measurable and evaluable indicators of certain biological states in normal and pathogenic processes, or possible pharmacologic responses to therapeutics [66]. More importantly, they can not only predict the occurrence or progression of diseases at the expression level, but also potentially indicate dynamic changes in biological processes or state [67].

Due to the critical role of telomeres and telomerase in the onset and development of cancer, an increasing number of studies have targeted telomeres and telomerase as biomarkers, resulting in the acquisition of many meaningful methods for detecting biomarker. These methods can be examined through blood, faeces, urine and other means without invasive testing [68] (Table 1). This is very easy to implement for many doctors and patients. These biomarkers can help doctors distinguish pancreatic cancer from other pancreatic diseases, and predict the incidence rate and prognosis of pancreatic cancer through telomere length and telomerase activity.

**Table 1:** Telomere and telomerase as Biomarker of pancreatic cancer.

Year	Biomarker	Tissue	Test purpose	Total sample size	Markers of pancreatic cancer	Refs
1997	telomerase activity	pancreatic tissue	diagnosis of pancreatic cancer	57	Positive telomerase activity may represent pancreatic cancer	[138]
1997	telomerase activity	pancreatic tissue	diagnosis of pancreatic cancer	45	Positive telomerase activity may represent pancreatic cancer	[70]
1999	telomerase activity	pancreatic juice	diagnosis of pancreatic cancer	16	Positive telomerase activity may represent pancreatic cancer	[139]
1999	telomerase activity	pancreatic tissue	pathologic staging	58	The higher the telomerase activity, the greater the possibility of pancreatic cancer	[72]
2000	telomerase activity	pancreatic juice	diagnosis of pancreatic cancer	31	Positive telomerase activity may represent pancreatic cancer	[140]
2000	telomerase activity	pancreatic tissue	diagnosis of pancreatic cancer	28	Positive telomerase activity may represent pancreatic cancer	[141]
2001	telomerase activity、expression of telomerase mRNA	pancreatic tissue	diagnosis of pancreatic cancer	86	Positive telomerase activity and high expression of telomerase mRNA may represent pancreatic cancer	[142]
2002	telomere length	pancreatic tissue	disease risk	76	Telomere shortening increases the incidence of pancreatic cancer	[36]
2004	telomerase activity	pancreatic tissue	diagnosis of pancreatic cancer	21	High telomerase activity may represent pancreatic cancer	[143]
2004	telomerase activity	pancreatic juice	diagnosis of pancreatic cancer	100	High telomerase activity may represent pancreatic cancer	[144]
2005	telomerase activity	pancreatic juice	diagnosis of pancreatic cancer	70	Positive telomerase activity may represent pancreatic cancer	[145]
2005	telomerase activity	pancreatic juice	diagnosis of pancreatic cancer	86	High telomerase activity may represent pancreatic cancer	[146]
2007	htert mRNA	bile	diagnosis of pancreatic cancer	20	The increased expression of hTERT mRNA may be associated with pancreatic cancer	[147]
2007	telomerase activity	pancreatic tissue	diagnosis of pancreatic cancer	21	High telomerase activity may diagnose pancreatic cancer	[148]
2008	telomerase activity	pancreatic juice	diagnosis of pancreatic cancer	17	High telomerase activity may diagnose pancreatic cancer	[149]
2008	telomere length、hTERT expression、telomerase activity	pancreatic tissue、pancreatic juice	pathologic staging	93	Short telomeres、high hTERT activity、High telomerase activity may increase the risk of pancreatic cancer	[33]
2012	telomere length	white blood cells in the blood	disease risk	1462	Short telomere length and ultra-long telomere length may increase the risk of pancreatic cancer	[150]
2015	Telomere length、 NTCR	pancreatic tissue	disease risk	219	The decrease of telomere length and NCTR may represent an increased risk of pancreatic cancer	[151]
2016	white blood cells telomere length、TLV、	white blood cells in the blood	disease risk	1800	Extremely short and long telomeres will increase the risk of pancreatic cancer, and the increase of TLV will increase	[86]
2017	telomere length	white blood cells in the blood	disease risk	1282	Shorter prediagnostic leukocyte telomere length was associated with higher risk of pancreatic cancer	[152]
2018	telomere fusions	pancreatic cyst fluid	pathologic staging	93	The appearance of telomere fusion may predict tissue carcinogenesis	[87]
2018	telomere length	pancreatic tissue, pleural effusion, ascites	pathologic staging	41	Short telomere length may be pancreatic cancer	[153]
2019	teloscore	pancreatic tissue	disease risk	6700	Low teloscore represents a higher risk of pancreatic cancer	[85]
2019	telomere length	white blood cells in the blood	survival	423	Longer telomeres may lead to longer survival time	[154]
2019	telomere length	white blood cells in the blood	disease risk	28346	Telomere length was positively associated with pancreatic cancer risk	[81]
2020	treatment-naïve LTL	white blood cells in the blood	disease risk	2919	Treatment-naïve short LTL is associated with a higher risk of PDAC,	[84]
2021	treatment-naïve LTL	white blood cells in the blood	overall survival	642	Shorter treatment-naïve LTL is associated with poorer overall survival	[82]
2022	p-hTERT	pancreatic tissue	poor prognosis	252	High p-hTERT expression predicts poor prognosis	[79]
2022	NCTR	pancreas tissue	pathologic staging	28	The NTCR showed a gradual decrease with increasing pathological grade of IPMNs	[35]

The sample size only includes the number of samples related to pancreatic lesions in the study and the control group.

*TLV* telomere length variation, *LTL* leukocyte telomere length, *p-hTERT* phosphorylation of hTERT at threonine 249, *NCTR* normalized telomere-centromere ratio.

In 1996, Mizumoto et al. compared human pancreatic cancer duct samples and the pancreatic cancer cell line MIA with normal pancreatic ducts and found that telomerase activity may be a specific marker for pancreatic duct cancer [69]. A year later, Tsutsumi et al. found an increase in telomerase activity in 32 out of 38 pancreatic ductal cancer samples compared to the surrounding tissue of the tumour [70]. However, no such change was found in the tissue surrounding the tumour. In 1998, Suehara et al. found that telomerase activity increased in the pancreatic juice of a patient, but no tumour was found in various examinations. After 19 months, the patient developed pancreatic cancer. This led the research team to suggest that telomerase activity in pancreatic juice could be a marker for the early diagnosis of cancer [71]. Subsequently, in 1999, researchers used telomerase activity as a biological indicator in patients with malignant and precancerous pancreatic cystic tumours, benign tumours and pseudocysts and found that its specificity was 67%, sensitivity was 100% and overall accuracy was 86% [72]. In the same year, cell experiments and clinical trials found that the higher the telomerase activity, the more invasive pancreatic cancer could be [73, 74]. As research deepened, in 2004 researchers found that the serum of pancreatic cancer patients could also be used for related tests to help diagnose pancreatic cancer [75]. In a study of 17 pancreatic cancer patients and 12 chronic pancreatitis patients, telomerase was found to be elevated in 88% of pancreatic cancer patients and 17% of chronic pancreatitis patients [76]. At present, it is not possible to distinguish whether elevated telomerase is caused by pancreatic cancer or chronic pancreatitis.

In addition to telomerase activity, the alteration of TERT also demonstrates its value as a biomarker. Research has shown that the hypermethylation region within the hTERT promoter is associated with upregulation of hTERT in cancers expressing hTERT, hence this region is called the TERT hypermethylation tumour region (THOR) [77]. A study in 2021 found that patients with higher levels of THOR methylation had shorter overall survival (OS) and recurrence-free survival (RFS) compared to patients with lower levels of THOR methylation, based on analysis of data from The Cancer Genome Atlas (TCGA) database [78]. In 2022, researchers also found that hTERT is phosphorylated at threonine 249 (Thr249) by the serine/threonine kinase CDK1 [79]. This phosphorylation is a molecular switch for hTERT to display RNA-dependent RNA polymerase (RdRP) activity, and the activity of hTERT RdRP can prevent the expression of some tumour suppressor genes, such as Forkhead box O4 (FOXO4), which can lead to pancreatic and liver cancer [79]. Through a study of 1523 cases of lung cancer, colon cancer, stomach cancer, pancreatic cancer, liver cancer, breast cancer and kidney cancer, it is concluded that the phosphorylation of hTERT at threonine 249(p-hTERT), which promotes the activity of RdRP, is a strong risk factor independent of TNM(Tumor Node Metastasis) staging and can be used as a biomarker for prognostic stratification of pancreatic cancer, lung cancer and other cancers [79].

In terms of telomere research, a study of 331 pathologies in 10 European countries showed that cell telomere length could not predict the incidence of pancreatic cancer [80]. In the study by Campa et al, 116 out of 26540 samples had been diagnosed with pancreatic cancer during an average follow-up of 12.8 years [81]. According to statistical analysis, longer leukocyte telomeres were significantly associated with the risk of pancreatic cancer [81]. An American study of 642 pancreatic cancer samples found that the telomere length of lymphocytes in white blood cells was negatively correlated with age at diagnosis [82]. The shorter telomeres, the shorter the survival, and the shorter the telomere, the higher the mortality [82]. Although telomere length is closely related to pancreatic cancer, statistical studies have shown that through gene discovery in 1500 pancreatic cancer patients and 1500 non-cancer patients, the genes regulating telomere length of leukocytes between the two groups cannot currently predict the incidence of pancreatic cancer, indicating that expression of genes regulating telomere length is related to many complex factors and needs further research [83]. Because first-line chemotherapy treatment affects the length of leukocyte telomeres, the research team also looked at the relationship between the length of leukocyte telomeres and the risk of pancreatic cancer in untreated patients [84]. The results showed that short leukocyte telomeres in untreated patients were associated with a high risk of pancreatic cancer [84]. These results suggest that when using leukocyte telomere length (LTL) as a biomarker, other confounding factors such as body size, diet, age and other stratified comparisons should be considered, which may help to provide more accurate predictive methods. One study suggested that the risk of pancreatic cancer could be assessed using the telomere score of lymphocytes. This study first looked at 11 genes that could affect telomere length and how many genes were associated with longer telomeres. A weighted calculation produces a number between 0 and 20. The higher the value, the longer the telomere, while the lower the value, the shorter the telomere. Finally, 2374 patients and 4326 controls showed that the higher the telomere score, the lower the risk of cancer [85]. Chinese scientists used telomere length variation (TLV) as an indicator to predict pancreatic cancer, which represents the heterogeneous telomere length at the ends of all chromosomes [86]. The white blood cells in the peripheral blood of 900 pancreatic cancer cases and controls were measured. It was found that an increase in TLV was associated with an increased risk of pancreatic cancer [86]. Another study predicted the occurrence of pancreatic cancer by detecting telomere fusion. This study suggested that telomere shortening would lead to telomere fusion and then telomerase activation, allowing pancreatic cancer cells to replicate indefinitely [87].

## Telomere and telomerase therapies

**Fig. 5:**
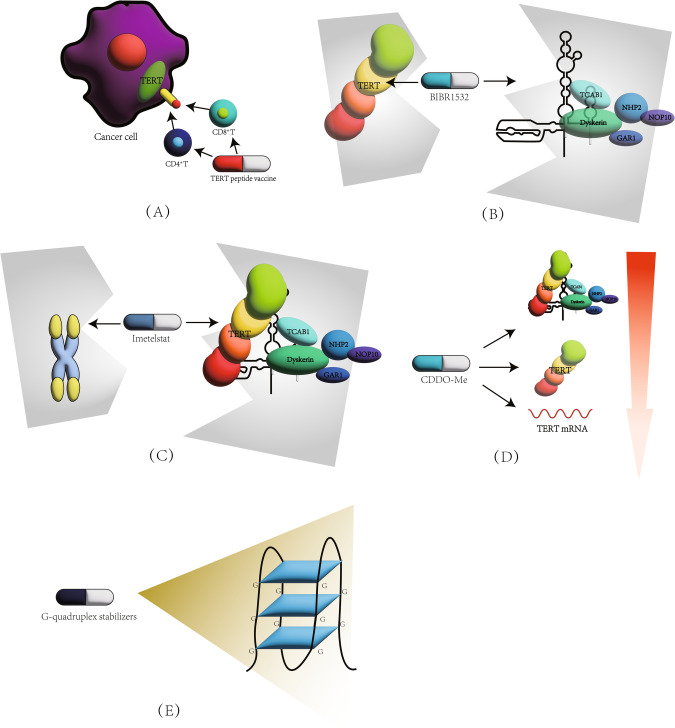
Common treatment methods targeting telomerase and telomerase. **A** Telomerase vaccines can recognize TERT peptides on the surface of cells with high TERT levels, thereby promoting the killing of CD4 + T and CD8 + T cells. The oncolytic virus can selectively replicate and lyse cells in cells activated by telomerase. **B** Small molecule inhibitor BIBR1532 can bind to TERT or TER, prevent the binding between TERT and TER, and inhibit the formation of telomerase. **C** The oligonucleotide inhibitor imetelstat can compete with telomeres and bind to TER, thereby preventing the binding between telomeres and telomerase. **D** CDDO Me can weaken hTERT mRNA, basal hTERT, phosphorylated hTERT, and methylation of hTERT promoter, thereby reducing telomerase activity. **E** G-quadruplex stabilizers can stabilize the G-quadruplex structure of telomeres, prevent DNA helicase from degrading them, and block the telomere elongation process.

As research into the relationship between telomeres, telomerase and various cancers gradually deepens, researchers suggest that treating cancer through telomerase inhibition is a promising research approach. Starting from this pathway may help us to understand and treat various cancers [88–91]. There are two main types of treatment using telomerase as a pathway: immunotherapy and telomerase inhibitor therapy [92–94] (Fig. 5). The drugs used for immunotherapy are the TERT peptide vaccine and oncolytic virus [92, 95]. Drugs used for direct inhibition of telomerase include oligonucleotide inhibitors, small molecule inhibitors and natural products [93, 96, 97]. Indirect inhibitors of telomerase are mainly divided into G-quadruplex stabilisers, nucleoside analogues and telomere uncapping agents [94, 98, 99].

### Immunotherapy

In immunotherapy, one of the TERT vaccines currently in clinical research for pancreatic cancer is called GV1001. This treatment targets cells with activated telomerase. As telomerase is often activated in pancreatic cancer cells, this treatment is likely to be one of the most important ways of treating pancreatic cancer in the future. In a study of 38 patients with inoperable pancreatic cancer in Norway, researchers used GV1001 and granulocyte macrophage colony-stimulating factor in patients, and the survival of 24 patients who responded was significantly prolonged, showing the drug’s therapeutic potential [100]. The efficacy of GV1001 is likely due to its ability to induce CD4 + T cells and CD8 + T cells in the immune system to mount an appropriate immune response, leading to a strong inflammatory response against tumours or tumour-draining lymph nodes, thereby achieving the effect of inhibiting tumours and prolonging patient survival [100]. Another UK study showed that the combination of GV1001 and chemotherapy did not improve the survival rate of locally advanced or metastatic pancreatic cancer patients, suggesting that the clinical potential of GV1001 needs further investigation [101]. At the same time, researchers have also begun to study the therapeutic strategy of combining telomerase vaccines with chemotherapy. In addition to the treatment of pancreatic cancer, GV1001 has also been developed for the treatment of other cancers, including melanoma, small cell lung cancer, prostate cancer, colorectal cancer, renal cell cancer, etc [102–106]. Its specific anticancer mechanisms include inhibition of HIF-1α、regulation of VEGF/VEGFR-2 signalling pathway that inhibits angiogenesis and selectively stimulates the Gαs/cAMP pathway that inhibits cancer cell proliferation, and so on. However, these cannot fully explain its mechanism and researchers need to do more research.

The other two vaccines used in pancreatic cancer research are INO-1400 and INO-1401. The difference between the two vaccines is that the former is modified to have 99% similarity to human TERT and the latter is further modified to have 95% similarity to human TERT than the former [107]. This modification may enhance the efficacy of the vaccine and help improve tolerance. The researchers treated pancreatic cancer with or without INO-9012. INO-1401 and INO-1401 are synthetic DNA plasmids encoding modified human telomerase protein, while INO-9012 consists of DNA plasmids encoding synthetic human IL-12 (p35 and p40 subunits) [107]. Following treatment, researchers observed an upregulation of CD38 on hTERT-specific CD4+ and CD8 + T cells in patients, as well as a survival benefit in patients [107].

In oncolytic virus research, there is a type of oncolytic virus called OBP-502 [108]. When telomerase is activated, its hTERT is often also in an activated state, and the activation of hTERT drives the expression of oncolytic virus E1A and E1B genes, allowing the oncolytic virus to selectively replicate in tumor cells, ultimately leading to tumor cell death. The virus then continues to replicate in surrounding cells, eliminating tumour cells. In cellular and animal studies, OBP-502 induced the release of ICD molecules from CT26 and PAN02 cells, leading to recruitment of CD8+ lymphocytes and inhibition of Foxp3+ lymphocyte infiltration into tumors [108]. Although its variant OBP-301 has entered clinical research, it may be some time before OBP-502 enters clinical practice.

Among the small molecule inhibitors, there is a non-competitive small molecule inhibitor called BIBR1532 [109]. It can disrupt the binding of TERT and TER, thereby reducing telomerase activity. In vitro experiments show that BIBR1532 can not only cause DNA damage to pancreas cancer samples and promote cancer stem cells(CSC) apoptosis, but also reduce the expression of TERT, prevent the number of CSCs in organoid culture medium and thus prevent them from forming spheres [48]. The augmented telomerase activity in CSCs is highly likely associated with the activation and mutation of TERT promoter, whereupon its activation or mutation leads to an increase in TERT activity, thus culminating in heightened telomerase function [49]. When BIBR1532 interacts with TERT within CSCs, it serves to inhibit telomerase activity, a mechanism that ultimately depletes the CSC [48]. Given the poor pharmacokinetic properties of this drug, it is difficult to say whether it can be used in clinical practice. However, in the future, new drugs may be developed and applied in clinical practice based on the mechanism of their pharmacological effects [95].

### Oligonucleotide inhibitor

In oligonucleotide inhibitor research, current research shows that ten strains of pancreatic cancer cells (L3.6pl, MiaPaCa2, HPAF, AsPC1, CD18, Panc1, Hs766T, CFPAC1, CAPAN1, CAPAN2) all respond to the oligonucleotide inhibitorimetelstat (GRN163L) [110]. Its mechanism of action is mainly to block the binding between telomeres and telomerase, thereby inhibiting the action of telomerase on telomeres [111]. However, for unknown reasons, sensitivity toimetelstat varies between different cell lines, resulting in varying degrees of telomerase inhibition. And when the drug was discontinued after it had worked, it was found that the drug continued to work for up to three weeks after discontinuation. This suggests that in future clinical practice, patients may be able to avoid taking the drug for a period of time after it has taken effect to reduce the occurrence of side effects [110]. Three weeks later, telomerase inhibition disappeared, and telomeres of pancreatic cancer cells were prolonged, γ- H2AX decreased again to the level when it was not administered, which confirmed the relationship among these factors in cancer. In addition, this experiment also showed the research potential ofimetelstat in the treatment of pancreatic cancer [110]. Research by Joseph et al. shows thatimetelstat can reduce the number of CSCs in both pancreatic and breast cancer cells, thereby inhibiting tumour cell growth and preventing tumours from progressing [112]. In the study of oesophageal cancer, long-term use of imetelstat therapy also inhibits telomerase activity, which not only reduces the proliferation of oesophageal cancer cells, but also induces increased DNA double-strand breaks (DSBs). after irradiation, which is helpful in the delivery of radiotherapy [113]. In studies of non-small cell lung cancer, imetelstat has an effect on different genetic characteristics and cell lines, and cells with shorter telomeres respond more quickly to imetelstat [114]. In clinical trials involving non-small cell lung cancer (NSCLC) patients treated with imetelstat, only individuals with shorter telomeres exhibited a propensity towards enhanced progression-free survival (PFS) and OS. Conversely, patients with longer telomeres did not exhibit statistically significant improvements in these clinical endpoints following imetelstat treatment. This suggests that there is still a long way to go in the clinical development of imetelstat [115].

### Inhibitors of natural compounds

In a study of natural compound, Deeb et al. found that the natural compound derivative methyl-2-cyano-3,12-dioxoene-1,9 (11) - dien-28-ester (CDDO-Me) can inhibit the proliferation of pancreatic cancer cells and promote their apoptosis at very low concentrations [116]. In in vitro experiments, CDDO-Me can attenuate hTERT mRNA, basal hTERT and phosphorylated hTERT, as well as telomerase activity, and inhibit hTERT promoter methylation [116]. The specific mechanism may be related to the core promoter of hTERT. In the experiment, it was found that CDDO-Me inhibited the activity of Sp1, c-Myc, and NF-κB in Panc-1 and MiaPaCa-2 cells [116]. These are binding sites of the core promoter of hTERT [117–119]. In the study by Deeb et al., it was also found that CDDO Me inhibits the protein levels of DNA methyltransferases DNMT1 and DNMT3a, leading to hypomethylation of the hTERT promoter [116]. Other studies have shown that the inhibition of telomerase activity by CDDO me can be mediated through ROS dependent pathways [120]. Gao et al.‘s study showed that CDDO-Me can inhibit tumor growth and prolong survival after tumor resection in animal experiments [121]. All this suggests that CDDO-Me is a potential drug for the treatment of pancreatic cancer in future, but clinical trials are still needed to verify this.

### G-quadruplex

G-quadruplex is a quadruplex consisting of four guanine and hydrogen bonds on DNA and RNA [122]. DNA helicase is required to break this structure prior to telomere elongation [123]. This structural change can upregulate or inhibit telomerase binding, and current research suggests that the stability of the G-quadruplex may help inhibit telomerase activity [124]. The 3’ end of telomeres binds to a protein known as POT, which plays a crucial role in preserving the stability of G-quadruplex and preventing its interaction with hTR [125]. This ultimately leads to the inhibition of telomerase activity [125]. Therefore, researchers in related fields are attempting to develop a ligand to make the G-quadruplex more stable. One of the G-quadruplex ligand drugs, BMSG-SH-3, has shown good therapeutic potential in animal studies of pancreatic cancer [126]. The mice did not lose weight during treatment, but tumour growth was reduced by 50% and telomerase activity was reduced by about 50%. And this drug is concentrated in tumours and the pancreas, and is less present in other organs [126]. This drug can either replace POT1 at the end of the telomere and binds to the single-stranded telomere DNA overhang, thereby inhibiting the telomerase activity [126, 127]. In addition, some G-quadruplex stabilisers such as naphthalene diimide, MM41, porphyrin-1 (cobalt-containing) and porphyrin-2 (palladium-containing) have also shown good experimental effects and clinical prospects [128–130].

### Potential issues

Although anti-telomerase cancer therapies have demonstrated encouraging research prospects, several potential concerns deserve careful consideration. Research findings suggest that the action of telomerase inhibitors on cancer cells may require a certain time frame to effectively shorten telomeres to a critical length, necessitating adequate periods of drug exposure for efficacious treatment [131]. However, during this interval, patients could potentially succumb to the progression of their cancer [132]. Consequently, there remains a compelling need to investigate more rapidly acting medications that can expedite the reduction in drug exposure times, thereby improving patient outcomes. Given that telomerase is typically not expressed in normal human somatic cells, and the fact that telomeres in normal stem cells are generally longer than those found in cancer cells, the anticipated impact of telomerase inhibitors on healthy cells is expected to be minimal [133]. Nonetheless, we cannot ignore the potential detrimental effects that telomerase inhibition may exert on certain cells, such as stem cells, which rely on telomerase activity during their proliferation process [134]. Particularly as the duration of treatment extends, the potential risks associated with telomerase inhibitors on normal cells may escalate concomitantly [135]. Additionally, telomeres serve as protective caps that maintain genomic stability in both normal and neoplastic cells. The suppression of telomerase activity in tumor cells results in telomere erosion, which could precipitate genomic instability within these malignantly transformed cells. This instability may further contribute to their carcinogenic progression. As such, the use of telomerase inhibitors necessitates careful and thorough assessment of the disease state to mitigate the risk of inadvertently promoting detrimental consequences. Although the disabling of telomerase activity leads to a brief slowdown in tumor growth. The therapeutic approach targeting telomerase inhibition may not be comprehensive, as telomere elongation is not solely dependent on the activation of telomerase. Telomeres can also be maintained through an alternative lengthening mechanism known as alternative lengthening of telomeres (ALT), which could potentially allow cancer cells to bypass the effects of telomerase inhibitors and sustain their telomere length. A subsequent resurgence in tumor proliferation occurred as these neoplasms adapted by adopting the ALT mechanism and aberrant transcriptional networks centering on mitochondrial biology and oxidative defense [136]. However, at present, the activation of ALT pathways in cancers appears relatively infrequent; the majority of malignancies primarily rely on modulating telomerase activity for telomere maintenance, with this ALT phenotype being observed in only approximately 10–15% of tumors [137]. Consequently, the preponderance of scientific attention has been directed towards the development and investigation of telomerase inhibitors as a therapeutic strategy.

## Conclusion

Telomeres and telomerase are closely linked to the occurrence and development of pancreatic cancer. The detection of telomerase can differentiate pancreatic cancer from other pancreatic diseases. Telomere length can also predict patient prognosis. Mutations in related genes can cause changes in telomeres and telomerase, reducing the body’s inhibition of cancer cells and promoting the proliferation and metastasis of pancreatic cancer cells. Many related physiological and pathological changes still need to be elucidated. The efficacy of telomerase inhibitor-based treatments also needs to be further investigated and their mechanisms of action explored. We can discover more accurate biomarkers based on research results, help researchers better design prospective studies, and pave the way for research into new diagnoses and treatments. This will allow medical researchers to develop better telomerase inhibitors and treat pancreatic cancer as safely as possible. In addition, research into telomeres and telomerase may not only help shorten the lifespan of tumours in humans, but also potentially extend the lifespan of patients with other diseases. This will have huge benefits for human health.
